# Utility of 3-(2-oxo-2*H*-chromen-3-yl)-1-phenyl-1*H*-pyrazole-4-carbaldehyde in the synthesis of novel 1,3,4-thiadiazoles or 1,3-thiazoles and study their cytotoxic activity

**DOI:** 10.1038/s41598-025-13664-2

**Published:** 2025-08-17

**Authors:** Anhar Abdel-Aziem, Abdou O. Abdelhamid

**Affiliations:** 1 Department of Chemistry, Faculty of Science (Girls), Al-Azhar University, Nasr City, Cairo, 11754 Egypt; 2 Department of Chemistry, Faculty of Science, Cairo University, Cairo, Egypt

**Keywords:** Coumarin, 1,3-thiazole, 1,3,4-thiadiazole, Hydrazonoyl halides, Cytotoxic activity, Biochemistry, Chemistry

## Abstract

One of the biggest causes of death around the world is cancer. Despite the development of a variety of chemotherapeutic drugs that stop excessive cell division, drug resistance remains a major obstacle to chemotherapy. It is consequently critical to develop and find new and effective anti-cancer agents in order to address the global threat. Owing to coumarins are a promising scaffold for anticancer agents, in this approach, we interested to synthesis new coumarins linked to either 1,3-thiazols or 1,3,4-thiadiazoles and examined their cytotoxic efficacy. Eleven of the newly prepared compounds were selected for the in vitro anticancer investigations against 60 human cancer cell lines at a single dose (10^− 5^M). Based on the results, we observed that compounds **3a**, **3b**, **6**, **8a**,** b** and **10b** were the most active towards different cancer cell lines where the growth inhibition percent (GIP) was up to 96%. Moreover, compound **6** had lethality effect against melanoma MDA-MB-435 and renal cancer A498 with growth percent − 47.47 and − 6.20. Also, the lethal effects were seen with compound **10b** where it exerted a value of − 27.79% toward melanoma MDA-MB-435.The highest GIP values 96.03% was recorded for compound **10a** against melanoma MDA-MB-435 cancer cell line.

## **Introduction**

Cancer is considered the second leading cause of death worldwide and it is expected to become the leading cause of death in the coming years^1^. According to the WHO, around 9.6 million deaths occurred in 2018 and are expected to reach 13.1 million by 2030^2,3^. Chemotherapy and immunotherapy are used for treatment of hematologic or metastatic malignancies. Radiotherapy and surgery are still used to treat local tumors. Despite the development of a variety of chemotherapeutic medicines that limit excessive cell division, drug resistance remains a key barrier to chemotherapy^4,5^. To combat this worldwide problem, researchers continue their efforts to discover new and effective anticancer therapies.

Coumarins are phytochemicals commonly found in plant roots, flowers, leaves, peels, seeds and fruits, as secondary metabolite^6–10^. Phytochemicals are bioactive components of plant-based foods e.g., fruits, vegetables, grains, and tea. Coumarin was first isolated in 1820 by Vogel from *tonka bean* in (*Dipteryx odorata*)^11,12^. The coumarins name originates from French word “*Coumarou*” for the *tonka bean*^13,14^. Scopoletin and esculetin are isolated from *Lycium chinense*^15^. Esculetin, esculin, fraxin and fraxetin are pharmacologically active ingredients isolated from *Cortex Fraxini*^10^. Esculetin has been widely used in expectorant, antitussive aspects^16^ and as anti-inflammatory, antioxidant, antibacterial^17^, and antitumour therapeutics.

**Fig. 1:**
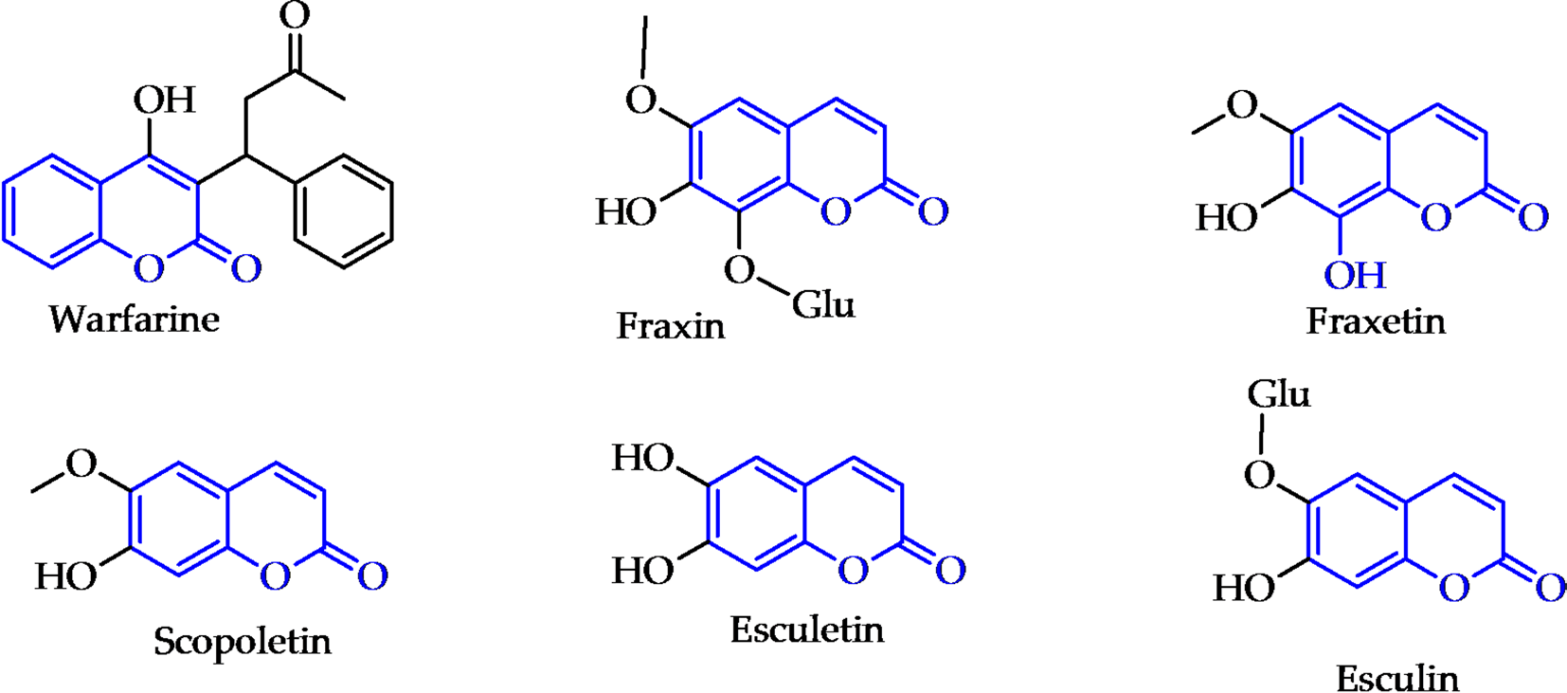
Naturally occuring coumarins used as drugs.

Coumarins are a key ingredient in perfumes, cosmetics and as industrial additives^18–20^. Furthermore, the stability and solubility of coumarins have piqued the interest of medical chemists, leading to their usage in medicine^21,22^. On the other hand, coumarin displays a broad range of biological and pharmacological activities such as anticancer^23–25^, antimicrobial^26–28^, antiviral^29–32^, antidepressant^33^, anti-coagulant^34^ antioxidant, anti-inflammatory, anti-HIV and antimalarial^35–39^. In addition, they exhibited antihyperlipidemic^40^, anti-Leishmanial^41^, antiprotozole^42^, antituberculosis^43^ and anti-Alzheimer activities^44^. On the other side, thiazoles are a class of heterocyclic compounds with a wide range of biological importance. They displayed anticancer and antimicrobial activities^45,46^. Also, 1,3,4-thiadiazoles are anticancer and antioxidant agents^47,48^. Depending on the above facts, this study aimed at the development of new coumarin derivatives as therapeutic approaches for cancer treatment.

## Results and discussion

### Chemistry

In our research program we utilized 3-(2-oxo-2*H*-chromen-3-yl)-1-phenyl-1*H*-pyrazole-4-carbaldehyde (**1**)^49^ as a precursor for synthesis of new heterocycles. Thus, condensation of compound **1** with benzyl or methyl hydrazinecarbodithioates **2a**,** b**^50,51^ in 2-propanol under stirring at room temperature afforded substituted-*2*-(3-(2-oxo-2*H*-chromen-3-yl)1-phenyl-1*H*-pyrazol-4-ylmethylene) hydrazine carbodithioates **3a**,** b** (Fig. 1). The hydrazinecarbodithioate derivatives **3a**,** b** was formed *via* nucleophilic attack of NH_2_ in compound **2** to the positively charged carbonyl carbon of CHO in compound **1** followed by elimination of water molecule.

The structure of the new derivatives **3a**,** b** was confirmed from their spectral data as well as chemical transformation. The IR spectrum of **3a**,** b** showed new absorption peaks at 3320 and 3385 cm^− 1^ assigned to NH group, in addition to a peak at 1275, 1284 cm^− 1^ for (C = S), respectively. ^1^H NMR spectrum of **3a** demonstrated five singlet signals at δ 3.00, 8.01, 9.99, 10.11 and 12.82 ppm assigned to SCH_3_, CH = N, CH-pyrazole, CH-coumarin and NH groups, consequently. ^1^H NMR spectrum of **3b** showed a new signals at δ = 4.38, 8.51, 9.03, 9.98 and 13.02 ppm corresponding to SCH_2,_ azomethine, CH protone of pyrazole, CH-coumarin and NH groups, consequently.

On the other side, a novel series of 1,3,4-thiadiazoles **5a-d** were synthesized *via* cyclocondensation of carbodithioate derivative **3a**,** b** with various hydrazonoyl halides **4a-e** under stirring in ethanol and triethylamine (Fig. 1). Confirmations the structure of new thiadiazoles was done by studying their spectroscopic data. ^1^H NMR of **5a** revealed triplet and quartet signals at δ 1.42 and 4.42 ppm for ethoxy group besid the rest aromatic protons. Its ^13^C NMR displayed signals at δ 14.19 (CH_3_), 63.08 (CH_2_), 116.71-158.51 (Ar-C) beside two signals at 159.80 and 164.96 ppm for two carbonyl groups. The mass spectrum of **5a** revealed a molecular ion peak at m/z = 562 (100%).

In addition, ^1^H NMR of **5b** recorded new signal at δ 2.87 for COCH_3_, 7.19–8.01 (m, 13H, ArH’s), 8.31(s, 1H), 8.45 (s, 1H), 8.84 (s, 1H), 8.98 (s, 1H). While, its ^13^C NMR demonstrated the existence of signals at δ 24.9 (CH_3_), 116.79-159.88 (Ar-C) and 162.52, 165.36 (2 C = O). The mass spectrum of **5a** revealed a molecular ion peak at m/z = 532 (, 100%).

**Fig. 2:**
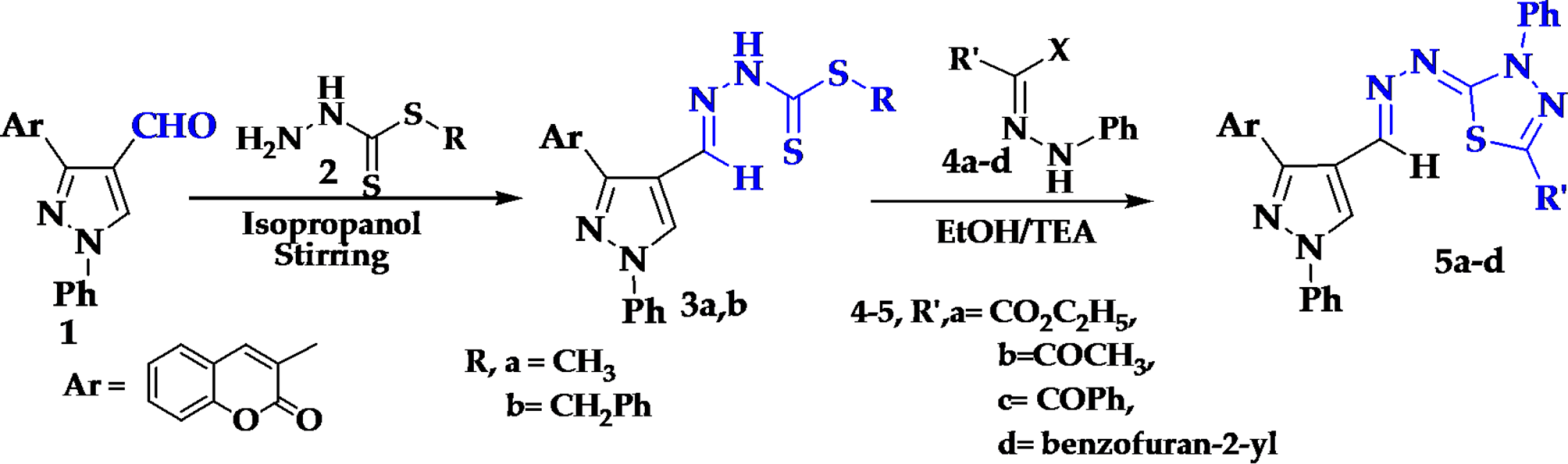
Synthetic pathway for synthesis of 1,3,4-thiadiazoles 5a-d.

**Fig. 3:**
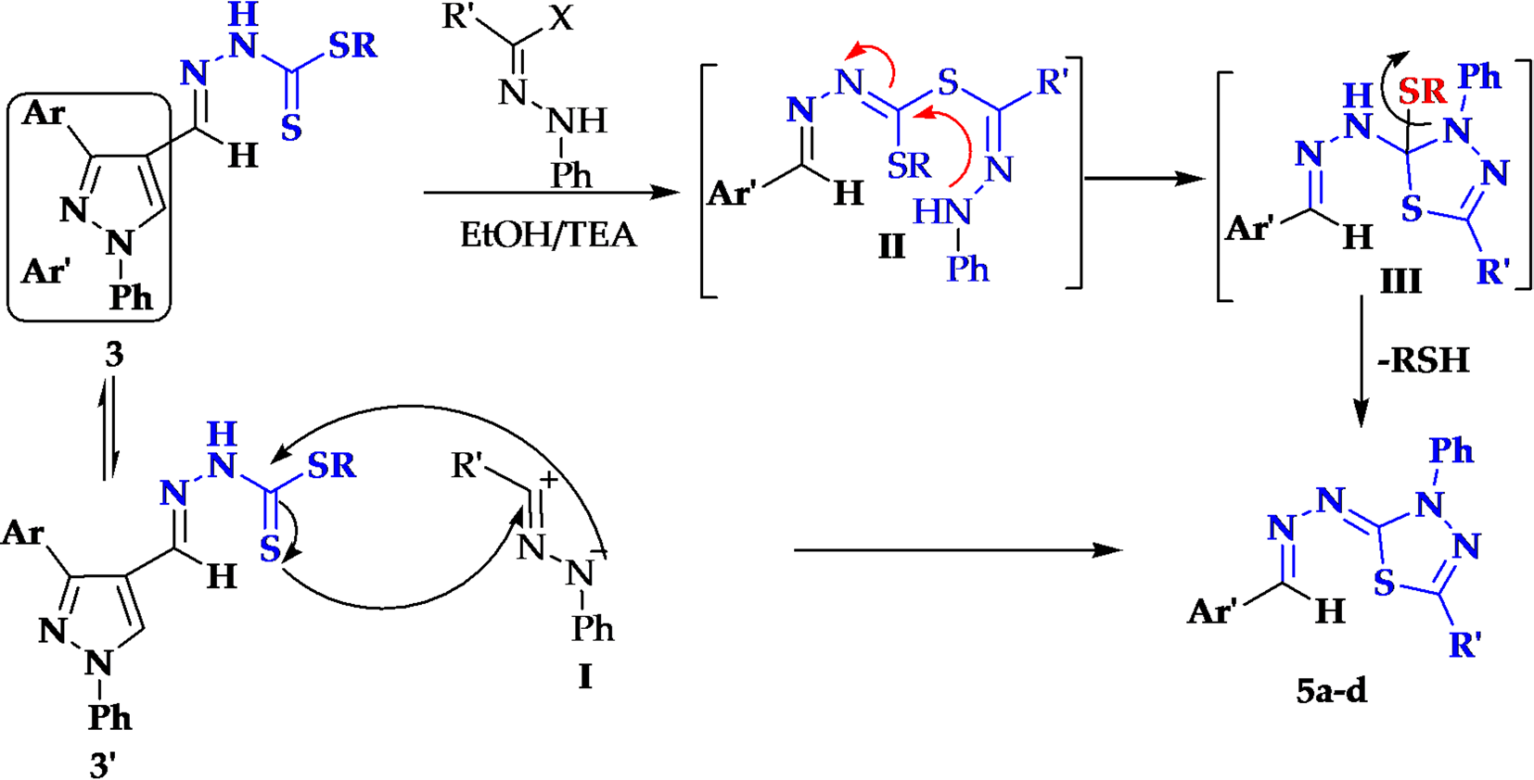
Mechanism of formation of 1,3,4-thiadiazoles 5a-d.

It is assumed that compounds **5a-d** can be formed through one of the two possible pathways^47^. The first pathway is the attack of SH group of compound **3 to** hydrazonoyl halide and losing of HX molecule to produce thiohydrazonate **II**, then intermolecular cyclization to give cyclo-adduct **III**.Finally, the intermediate **III** losses either benzyl thiol or methylthiol molecule to give thiadiazoles **5a-d**. In the second pathway the nitrileimine **I** [prepared in situ from **4a-d** with triethylamine] underwent 1, 3-dipolar cycloaddition to the double bond of C = S of **3’** and give **II** directly (Figs. 2 and 3).

**Fig. 4:**
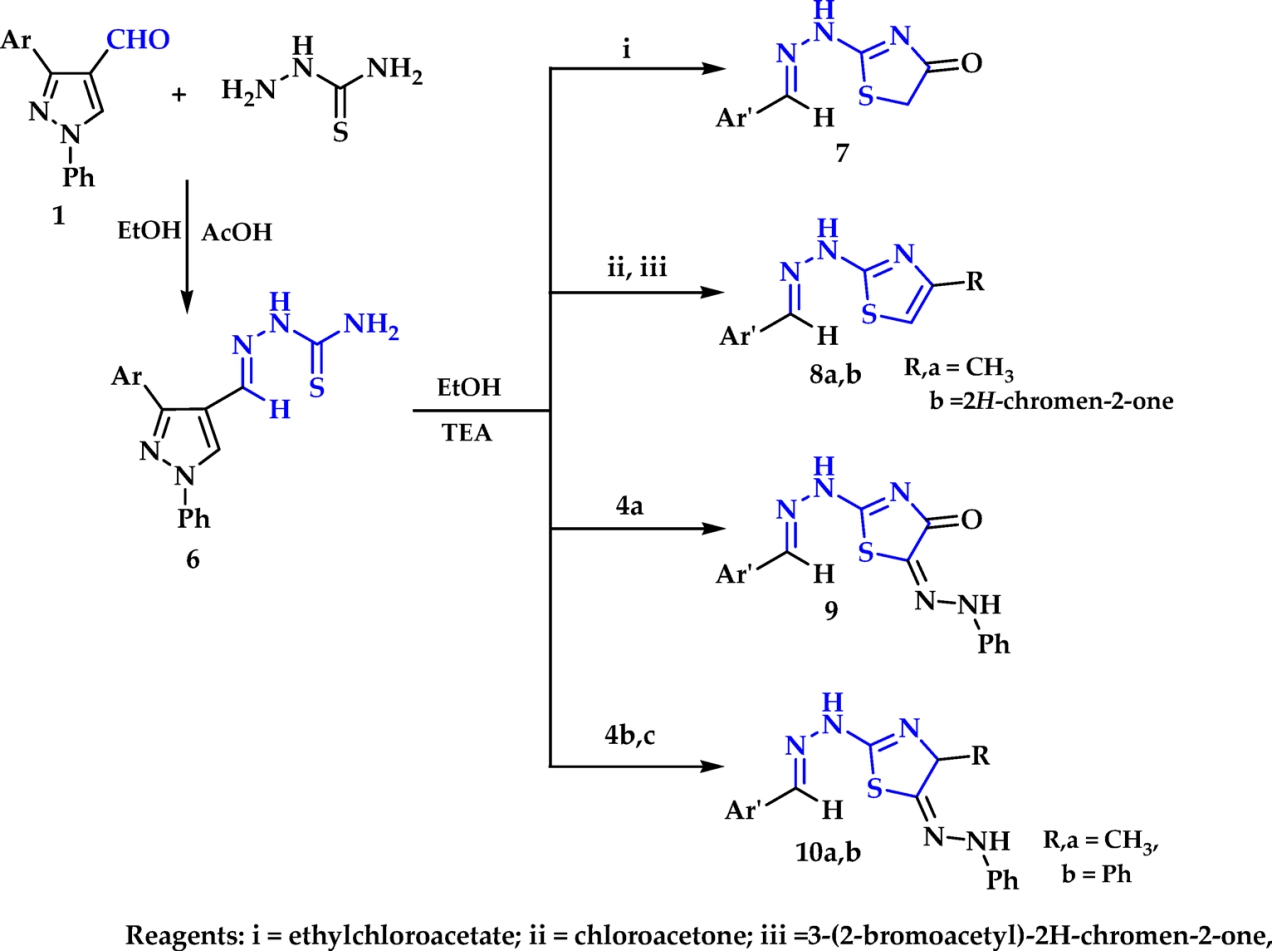
Synthetic pathway for synthesis of thiazoles and thiazolinones.

Moreover, compound **1** was condensed with thiosemicarbazide to furnish thiosemicarbazone **6**. Cyclocondensation of thiosemicarbazone **6** with α-haloketone or α -haloester was reported herein. Hence, reaction of **6** with ethyl chloroacetate, chloroacetone or 3-(2-bromoacetyl)-2*H*-chromen-2-one^52^ in ethanol and triethylamine as a catalyst under reflux afforded 1,3-thiazolinone **7** and 1,3-thiazoles **8a**,** b**, respectively (Fig. 4). The IR of compound **7** displayed an absorption peaks at 3424 cm^− 1^ for NH function, 1722 and 1676 cm^− 1^ due to (2 C = O). Besides, its mass spectrum revealed a molecular ion peak at m/z = 429 (33.9%) and a base peak at 314 (100). In addition, the ^1^H NMR of compound **7** recorded signals at δ 2.39 ppm for methyl group, 7.11-7.91ppm multiplet for nine aromatic protons, in addition to, five singlet signals at 8.06, 8.21, 8.79, 9.10 and11.12 ppm. Its mass spectrum showed a molecular ion peak at *m/z* = 427 (37.96%) and 114 (100).

On the other hand, the activity of thiosemicarbazone **6** towards some hydrazonoyl halides was studied. Thus, cyclocondensation reaction of thiosemicarbazone **6** with hydrazonoyl halides^53,54^ **4a-c** in ethanol and triehylamine gave thiazolinone **9** and phenylazothiazoles **10a**,** b** (Scheme 3). The structure of these products can be confirmed by elemental analysis besides spectral data. ^1^H NMR of **10a** showed signals at δ 2.45ppm for methyl protons, 7.38–7.65 (m, 10 H), triplet at δ 7.85ppm for one proton with *J* = 7.7 Hz, two doublets at δ 7.95 and 8.39ppm for two protons with *J* = 7.6 and 8.6 Hz. In addition to four singlet signals at δ 8.00, 8.21, 8.93 and 9.15 ppm for aromatic H and singlet signals at δ 11.33 ppm assigned to NH proton. The structure was further confirmed by mass spectrum and showed a molecular ion peak at m/z = 531 (49.33) and 77 (100%).

### Pharmacological screening

The newly synthesized compounds **3a**,** b**, **5a-d**, **6**, **9a**,** b** and **10a**,** b** were chosen by the National Cancer Institute (NCI), Bethesda, Maryland, USA to investigate their cytotoxic activity. The selection of these compounds was based on their chemical structure, anticancer activity, SAR studies and computer modeling techniques. The screening were done against 60 different human tumor cell lines, representing leukemia, melanoma and cancers of the lung, colon, breast, brain, ovary, kidney and prostate. The tested compounds were subjected to screening at single dose concentration of 10^− 5^M. The Inhibition growth percent (IGP) for each of the tested compounds against the cancer cell lines are shown in Table 1.From the results it was clear that, compound **3a** displayed high potency against most tested cancer cell lines, exerting IGP values ranging from 41.72 to 79.20%), and was moderately active against few cells with GIP values ranging from 20.87 to 38.09%. Compound **3b** showed moderate to high potency toward all leukemia cell lines with GIP (35.90-63.42%) except HL-60(TB) cell line (GIP; = 22.66%). It also showed good activity against non-small cell lung cancer NCI-H460 (GIP = 61.59%), colon cancer HCT-116, HCT-115 (GIP = 62.58 and 53.11%), CNS cancer U251 (GIP = 53.06%), renal cancer UO-31(GIP = 40.41%), as well as breast cancer MCF7 (GIP = 43.04%). In addition to moderate activity with non-small cell lung cancer HOP-92 (GIP = 35.20%), CNS cancer SF-268 (GIP = 27.12%), ovarian cancer OVCAR-8 (GIP = 28.73%) and prostate cancer PC-3 (GIP = 29.17%). It was observed that conversion of compounds **3a** and **3b** to 1,3,4-thiadiazole substituted at C2 led to remarkable decrease in the anticancer activity in all cancer cell lines as in compound **5a-d**. Compound **5a** with ester moiety in 2-position of thiadiazole ring was moderately active only with renal cancer UO-31 (GIP = 25.61%) and breast cancer MCF7 (GIP = 25.11%).Whereas, compound **5b** with acetyl moiety showed weak activity in almost all cancer cell lines. However, compound **5c** and **5d** demonstrated moderate activity against leukemia K-562 and SR (GIP = 21.79–31.52%). Besides, moderate activity toward non-small cell lung cancer HOP-92 (GIP = 28.51 and 26.77%), renal cancer UO-31 (GIP = 27.30 and 31.53%). Compound **5c** with benzoyl group attached to 2-position of thiadiazole ring exhibited moderate activity against leukemia CCRF-CEM, colon cancer (HCT-116, HCT-15), CNS cancer U251 beside breast cancer MCF7 with GIP = 21.56%, 22.01, 31.83%, 30.04% and 21.06%, respectively. It was noticed that thiosemicarbazone derivative **6** exerted remarkable activities against almost all cancer cell lines with inhibition growth percent ranging from 91.52 to 30.27%. **It exerted lethality against melanoma MDA-MB-435 and renal cancer A498 with growth percent − 47.47 and − 6.20.** On the other hand, conversion of compound **6** to thiazoles **8a**,** b** resulted in remarkable decrease in the anticancer activity. With respect to Leukemia K-562, SR cell lines compound **8b** with coumarin moiety attached to thiazole ring was the most active (IGP = 32.24, 39.00%) than compound **8a** with methyl group (IGP = 20.20, 26.91%). In addition, compound **8b** was moderately active against Colon Cancer HCT-15, SW-620 (IGP = 28.85, 30.31%) and Melanoma M14, MDA-MB-435 (IGP = 45.30, 43.80%). For Renal Cancer UO-31, compounds **8a** and **8b** were moderate active (IGP = 31.51, 28.91%), only compound **8b** was active against Breast Cancer MCF7 (IGP = 35.21%). Furthermore, compounds **10a** and **10b** with displayed potent to strong activity against leukemia cancer cell lines, exerting IGP values ranging from 30.15 to 78.14%), in addition, compound **10b** was strong active against non-small cell lung cancer A549/ATCC, NCI-H522 (IGP = 49.87, 58.51%) and colon cancer (IGP = (68.46–36.25%). Both compounds **10** and **10b** were most active with CNS cancer SNB-75 (IGP = 49.57, 73.39%), only compound **10b** was most active with CNS cancer U25 (IGP = 52.39%).The highest activity was observed with melanoma MDA-MB-435 where the IGP values was 96.03% for compound **10a**, the lethal effects were showend with compound **10b** with growth value − 27.79%. Finally, compound **10b** was most potent against breast cancer 38.23–55.75%.

**Table 1:** The cytotoxic screening data eleven compounds against Sixty human tumor cell lines at a single dose assay (10^−^5 M concentration) as Inhibition growth percent (IGP) %.

Cancer cell /Compd No.		3a		3b		5a		5b		5c		5d		6		8a		8b		10a		10b	
*Leukemia*																							
CCRF-CEM HL-60(TB) K-562 MOLT-4 RPMI-8226 SR		**64.13** **38.09** **61.21** **61.21** **57.49** **65.60**		**49.8** 22.66 **63.42** **35.90** **40.99** **50.91**		– – – – – –		– – – – – 11.95		21.56 – **31.52** 19.17 18.22 23.08		– – 21.79 – – **27.35**		**46.29** **79.6** **82.86** **66.61** **66.61** **87.78**		11.04 – 20.20 17.63 8.78 26.91		16.95 **30.15** **32.24** 11.35 20.95 **39.00**		– 10.99 **65.96** 14.95 10.75 **70.06**		**30.28** **41.87** **78.32** **46.48** **36.87** **78.14**	
*Non-Small Cell Lung Cancer*																							
A549/ATCC EKVX HOP-62 HOP-92 NCI-H226 NCI-H23 NCI-H522M NCI-H460 NCI-H522		22.16 **29.37** 14.74 **30.44** **34.45** 24.72 **36.51** **79.20** **26.88**		14.8 12.61 – **35.20** – – 11.17 **61.59** 15.67		– – – 17.68 – – – – –		– – – – – – – – –		16.56 – – **28.51** – – – 10.87 –		– – – **26.77** – – – – 12.05		**53.59** 24.67 27.68 20.31 − 21.83 10.73 **78.95 63.53**		10.73 – – – – – – – 13.84		11.02 – – – – 16.06 – – 20.93		– – **34.00** – – – – – **33.82**		**49.87** 17.70 **28.42** 20.28 – 11.89 – 26.54 **58.51**	
*Colon Cancer*																							
COLO 205 HCC-2988 HCT-116 HCT-15 HT29 KM12 SW-620		– 13.03 **69.1** **68.91** **30.54** **54.94** **49.42**		**–** **–** **62.58** **53.11** 19.79 21.09 **34.05**		– – – – – – –		– – – – – – –		**–** **–** 22.01 **31.83** – – –		– – 10.25 12.19 – – –		**35.63** 11.61 **72.78** **71.76** **85.28** **69.15** **77.29**		– – – – – – –		– – 7.19 **28.85** – 17.48 **30.31**		– – 12.64 **43.95** 14.84 36.25 21.23		**27.23** 14.35 **43.09** **58.11** **68.46** **55.31** **56.57**	
*CNS Cancer*																							
SF-268 SF-295 SF-539 SNB-19 SNB-75 U251		**45.31** **42.09** 21.98 **34.30** **31.00** **58.60**		**27.12** 13.69 11.38 – 10.96 **53.06**		– – – – – –		– – – – – –		12.17 – – – – **30.04**		– – – – – 16.93		**33.87 51.86** **42.82** **43.44** **91.52** **54.82**		– – – – – –		11.87 – – – – 11.03		– 18.36 12.34 – **49.57** –		20.69 **29.06** 11.54 14.95 **73.39** **52.39**	
*Melanoma*																							
LOX IMVI MALME-3 M M14 MDA-MB-435 SK-MEL-2 SK-MEL-29 SK-MEL-5 UACC-257 UACC-62		**58.92** 20.87 **38.69** **33.70** – 10.55 **31.88** 10.63 **41.77**		21.94 – 21.78 14.14 – – – – 19.67		– – – – – – – – –		– – – – – – – – –		13.80 – – – – – – – 11.90		– – – 18.89 – – – – –		**53.70** **51.21** **77.89** **–47.47** **60.78** **30.27** **53.75** **28.51** **65.78**		– – – – – – – – 21.44		– 19.79 **45.30** **43.80** – – 14.24 15.71 13.51		10.54 10.38 **29.26** **96.03** – 12.39 – – **29.02**		**29.96** 23.00 **54.57** **− 27.79 35.08** 11.01 20.27 13.53 **40.20**	
*Ovarian Cancer*																							
GROV1 OVCAR-3 OVCAR-4 OVCAR-5 OVCAR-8 NCI/ADR-RES SK-OV-3		**51.32** **35.15** **38.11** 15.84 **41.72** **50.66** –		19.36 14.50 14.21 – **28.73** 14.52 –		– – – – 10.00 – –		– – – – – – –		– – – – – 11.15 –		– – – – – – –		**53.26** **65.61** 20.81 11.91 **35.49** **51.42** 16.83		– – – – – –		7.23 – 17.96 – 19.24 12.68 –		10.91 – 14.61 – – 12.01 –		18.31 **42.89** 15.35 – 22.11 **36.26** 17.23	
*Renal Cancer*																							
786-0 A498 ACHN CAK1-1 RXF 393 SN12C TK-10 UO-31		**31.46** – **24.86** **34.22** **29.28** **26.64** **23.07** **63.33**		11.03 **–** **–** 13.38 **–** **–** 15.39 **40.41**		– – 10.58 – – – **25.61**		– – – 11.46 – – – 16.00		– – – – – – – **27.30**		– – – 20.90 – – – **31.53**		**37.60** **–6.20** 22.59 **55.89** 22.02 **36.89** 22.76 **49.30**		**–** **–** **–** **–** **–** **–** **–** **31.51**		– – – 14.18 – – – **28.91**		– 23.00 – **30.30** – – – **29.33**		– **29.79** – **37.16** – – 26.57 –	
*Prostate Cancer*																							
PC-3 DU-145		**36.00** **26.84**		**29.17** –		– –		– –		**–** **–**		**–** **–**		**32.51** 14.72		– –		– **23.73**		14.16 –		17.79 23.24	
*Breast Cancer*																							
MCF7 MDA-MB-231/ATCC HS 578T BT-549 T-47D MDA-MB-468		**56.11** 22.07 **29.62** **36.96** **33.76** **29.45**		**43.04** 22.31 – 15.00 21.59 –		– **25.11** – – 10.48		– 17.38 – – 10.95 –		21.06 19.68 – – 12.82 –		16.85 13.77 – – – –		**66.70** **65.65** **35.61** **57.46** **52.19** **56.66**		12.40 10.87 – – – –		**35.21** 12.23 – – 18.22 –		**31.71** 12.27 – 21.73 16.76 10.27		**55.75 31.25** 12.48 **38.23 42.62 51.45**	

Bold values represent the highest results in each cell; – Refers to < IGP (10%).

### Experimental

All the utilized reagents and solvents were of commercial grade. Melting points may be uncorrected and were determined on the digital melting point apparatus (Electro thermal 9100, Electro thermal Engineering Ltd, serial No. 8694, Rochford, United Kingdom). Infrared (IR) spectra were acquired on a Nicolet is10 FTIR instrument within the wavenumber range of 4000–400 cm^− 1^. Elemental analyses were performed on CHNS-O analyzer (Perkin-Elmer, USA). A Bruker Avance spectrometer (Bruker, Germany) was used to measure the ^1^H and ^13^C NMR spectra of the synthesized compounds at 400 and 101 MHz, respectively, using TMS as the internal standard. The following symbols were used for pointing to hydrogen coupling patterns; (s) singlet, (d) doublet, (t) triplet, (q) quartet and (m) multiplet. Chemical shifts were defined as parts per million (ppm) relative to the solvent peak. Also the mass spectra of the new compounds were measured with Electrospray Ionization Mass Spectrometry (ESI-MS).

### Synthesis of hydrazinecarbodithioates 3a and 3b

A solution of **1** (3.16 g, 10 mmol) and alkyl hydrazinedithioates **2a** or **2b** (10 mmol) in 10 mL of 2-propanol was stirred for 30 min. The white solid obtained was filtered and recrystallized from DMF affording **3a** and **3b**, respectively.

### Methyl-2-(3-(2-oxo-2 H-chromen-3-yl)-1-phenyl-1 H-pyrazol-4-yl)methylene) hydrazine-1-carbodithioate (3a)

White powder; Yield: 85%; m.p: 183 –85 °C; IR (KBr) cm^− 1^: 3320 (NH), 3090, 2925 (CH), 1706 (C = O), 1590 (C = N), 1492 (C = C), 1275 (C = S); ^1^H NMR (DMSO-*d*_*6*_) δ3.0 (s, 3 H, SCH_3_), 7.25–7.70 (m, 9 H, Ar-H), 8.01 (s, 1H, CH-pyrazole, 9.99 (s, 1H, CH-coumarin), 10.11 (s,1H, CH = N), 12.82 (s, 1H, NH); ^13^C NMR (DMSO-*d*_*6*_) δ 20.9 (SCH_3_), 116.70,, 119.80, 120.73, 122.33, 122.95,, 123.63, 127.44, 127.65, 128.28, 128.94, 129.28, 129.30, 129, 59, 131.78, 132.29, 138.00, 139.30, 142.57, 146.80, 149.44, 151.72, 154.13, 159.81 (Ar-C), 161.52 (C = O), 195 (C = S); MS *m/z* (%): 420 (M^+^, 2). Anal. calcd. for C_21_H_16_N_4_O_2_S_2_(420.51): C, 59.98; H, 3.84; N, 13.32; S, 15.25%. Found: C, 59.92; H, 3.89; N, 13.39; S, 15.32%.

### Benzyl-2-(3-(2-oxo-2 H-chromen-3-yl)-1-phenyl-1 H-pyrazol-4-yl)methylene) hydrazine-1-carbodithioate (3b)

White powder; Yield: 80%; m.p: 170 –71 °C; IR (KBr) cm^− 1^: 3385 (NH), 3067, 2920 (CH), 1706 (C = O), 1603 (C = N), 1572 (C = C), 1284 (C = S); ^1^H NMR (DMSO-*d*_*6*_) δ 4.38 (s, 2 H, SCH_2_), 6.99–7.50 (m, 14 H, Ar-H), 8.51 (s, 1H, CH-pyrazole), 9.03 (s, 1H CH-coumarin), 9.98 (s,1H, CH = N), 13.02 (s,1H, NH); ^13^C NMR (DMSO-*d*_*6*_) δ 39.9 (SCH_2_), 117.70- 158.81 (Ar-C), 162.52 (C = O), 200 (C = S); MS *m/z* (%): 496 (M^+^, 2.02), 495 (M^+^ - 1, 4.43), 494 (M^+^ -2, 14.1), 91 (100). Anal. calcd. for C_27_H_20_N_4_O_2_S_2_(496.6): C, 65.30; H, 4.06; N, 11.28; S, 12.91%. Found: C, 65.36; H, 4.12; N, 11.30; S, 12.97%.

### Synthesis of 1,3,4-thiadiazoles 5a-d

A mixture of alkyl hydrazinedithioates **3a** or **3b** (5 mmol) and the appropriate hydrazonoyl halides **4a-d** (5 mmoles) in 15 mL of ethanol was stirred. Triehylamine (0.075 mL, 0.005 mol) was added to this mixture with continues stirring. The colored precipitate obtained after 10 min was collected and recrystallized by DMF/EtOH affording the desired products **5a-d**, respectively.

### Ethyl-5-(3-(2-oxo-2 H-chromen-3-yl)-1-phenyl-1 H-pyrazol-4-yl)methylene) hydrazono)-4,5-dihydro-4-phenyl-1,3,4-thiadiazole-2-carboxylate (5a)

Yellow powder; Yield: 85%; m.p: 190 –91 °C; ^1^H NMR (CDCl_3_), δ 1.42 (t, 3 H, *J* = 7*Hz*, CH_3_), 4.42 (q, 2 H, *J* = 7*H*z, CH_2_), 7.29–7.62 (m, 6 H, Ar-H), 7.79 (d, 2 H, *J* = 8*H*z, Ar-H), 7. 92 (d, 2 H, *J* = 8*H*z, Ar-H), 7.97 (d, 2 H, *J* = 8*H*z, Ar-H), 8.42 (s,1H, CH = N), 8.50 (s, 1H, CH-pyrazole), 8.89, 10.09 (2s, 2 H, CH-coumarin); ^13^C NMR (CDCl_3_), δ 14.19 (CH_3_), 63.08 (CH_2_), 116.71, 119.36, 119.63, 122.73, 124.23, 124.58, 127.18, 127.44, 127.67, 128.33, 128.88, 129.25, 129.37, 129.58, 131.96, 132.33, 138.93, 139.42, 142.44, 143.03, 143. 80, 146.79, 148.28, 154.16, 158.51 (Ar-C), 159.80, 164.96 2(C = O); MS *m/z* (%): 562 (M^+^, 100). Anal. calcd. for C_30_H_22_N_6_O_4_S (562.6): C, 64.05; H, 3.94; N, 14.94; S, 5.70%. Found: C, 64.10; H, 3.99; N, 14.88; S, 5.76%.

### 2-Acetyl-5-(3-(2-oxo-2 H-chromen-3-yl)-1-phenyl-1 H-pyrazol-4-yl)-methylene)hydrazono)-4,5-dihydro-4-phenyl-1,3,4-thiadiazole (5b)

Yellow powder; Yield: 84%; m.p: 195 –96 °C; IR (KBr) cm^− 1^: 3062, 2919 (CH), 1689 (C = O), 1607 (C = N), 1595 (C = C); ^1^H NMR (CDCl_3_), δ 2.87 (s, 3 H, CH_3_), 7.19–8.01 (m, 13H, Ar-H), 8.31(s,1H, CH = N), 8.45 (s, 1H, CH-pyrazole), 8.84 (s, 1H, CH-coumarin), 8.98 (s, 1H, CH-coumarin); ^13^C NMR (CDCl_3_), δ 24.9 (CH_3_), 116.79, 119.59, 119.85, 121.73, 122.48, 123.95,, 124.63, 127.44, 127.68, 128.28, 128.94, 129.28, 129.34, 129, 59, 131.84, 132.29, 138.00, 139.39, 142.57, 143.95, 146.80, 148.44, 150.72, 154.13, 159.88 (Ar-C), 162.52, 165.36 (2 C = O); MS *m/z* (%): 534 (M^+^ + 2, 12.03), 533 (M^+^ + 1, 35.7), 532 (M^+^, 100). Anal. calcd. for C_29_H_20_N_6_O_3_S (532.57): C, 65.40; H, 3.79; N, 15.78; S, 6.02%. Found: C, 65.46; H, 3.72; N, 15.70; S, 6.09%.

### 2-Benzoyl-5-(3-(2-oxo-2 H-chromen-3-yl)-1-phenyl-1 H-pyrazol-4-yl)- methylene)hydrazono)-4,5-dihydro-4-phenyl-1,3,4-thiadiazole (5c)

Red powder; Yield: 84%; m.p: 220 –22 °C; IR (KBr) cm^− 1^: 3060, 2924 (CH), 1721 (C = O), 1601 (C = N), 1545 (C = C); ^1^H NMR (CDCl_3_), δ 7.18–7.78 (m, 11 H, Ar-H), 7.93–8.10 (m, 8 H, Ar-H), 8.88 (s,1H, CH = N), 9.39 (s, 1H, CH-pyrazole), 10.13 (s, 1H, CH-coumarin); ^13^C NMR (CDCl_3_), δ 116.67-160.68 (Ar-C), 162.70, 164.45 (2 C = O); MS *m/z* (%): 596 (M^+^ + 2, 12.8), 595 (M^+^ + 1, 38.48), 594 (M^+^, 96.70), 77 (100). Anal. calcd. for C_34_H_22_N_6_O_3_S (594.64): C, 68.67; H, 3.73; N, 14.13; S, 5.39%. Found: C, 68.61; H, 3.79; N, 14.20; S, 5.32%.

### 2-(Benzofuran-2-yl)-5-(3-(2-oxo-2 H-chromen-3-yl)-1-phenyl-1 H-pyrazol-4-yl)- methylene)hydrazono)-4,5-dihydro-4-phenyl-1,3,4-thiadiazole (5d)

Red powder; Yield: 84%; m.p: 270 –71 °C; IR (KBr) cm^− 1^: 3136 (CH), 1733, 1658 (C = O), 1606 (C = N), 1570 (C = C); MS *m/z* (%): 634 (M^+^, 0.73). Anal. calcd. for C_36_H_21_N_6_O_4_S (634.66): C, 68.13; H, 3.49; N, 13.24; S, 5.05%. Found: C, 68.20; H, 3.42; N, 13.30; S, 5.12%.

### Synthesis of compounds 1,3-thiazoles 7–10

A mixture of thiosemicarbazone **6** (5 mmol) and the appropriate hydrazonoyl halide **4a–c**, α haloketone or α-haloester (5 mmol, each) in ethanol containing trimethylamine was heated under reflux for 2 h. The precipitate that separated on hot was filtered and recrystallized from DMF/EtOH giving the desired products **7–10**.

### 2-{N’-[3-(2-Oxo-2 H-chromen-3-yl)-1-phenyl-1 H-pyrazol-4-ylmethylene]-hydrazino}-thiazol-4-one (7)

Yellowish white powder; Yield: 75%; m.p: 250 –51 °C; IR (KBr) cm^− 1^: 3424 (NH), 3098, 2936 (CH), 1722, 1676 (C = O), 1607 (C = N), 1573 (C = C); MS *m/z* (%): 431 (M^+^ + 2, 2.97), 430 (M^+^ + 1, 10.41), 429 (M^+^, 33.9), 314 (100). Anal. calcd. for C_22_H_15_N_5_O_3_S (429.45): C, 61.53; H, 3.52; N, 16.31; S, 7.47%. Found: C, 61.63; H, 3.60; N, 16.41; S, 7.39%.

### 3-{4-[(4-Methyl-thiazol-2-yl)-hydrazonomethyl]-1-phenyl-1 H-pyrazol-3-yl}-chromen-2-one (8a)

Black powder; Yield: 70%; m.p: 260 –62 °C; IR (KBr) cm^− 1^: 3214 (NH), 3136, 2975 (CH), 1734, 1697 (C = O), 1608 (C = N), 1598 (C = C); ^1^H NMR (DMSO-*d*_*6*_) δ 2.39 (s, 3 H), 7.11–752 (m, 6 H, Ar-H), 7.60–7.91 (m, 3 H, Ar-H), 8.06 (s, 1H, CH = N), 8.21 (s, 1H-CH-thiazole), 8.79 (s, 1H, CH-pyrazole), 9.10 (s, 1H, CH-coumarin), 11.12 (s, 1H, NH); ^13^C NMR (DMSO-*d*_*6*_) δ 22 (CH_3_), 116.76- 157.81 (Ar-C), 162.52 (C = O); MS *m/z* (%): 429 (M^+^ + 2, 3.2), 428 (M^+^ + 1, 11.04), 427 (M^+^, 37.96), 114 (100). Anal. calcd. for C_23_H_17_N_5_O_2_S (427.48): C, 64.62; H, 4.01; N, 16.38; S, 7.50%. Found: 64.70; H, 4.10; N, 16.48; S, 7.61%.

### 3-{4-[(4 –(2 H-chromen-2-one)-thiazol-2-yl)-hydrazonomethyl]-1-phenyl-1 H-pyrazol-3-yl}-chromen-2-one )8b(

Brown powder; Yield: 72%; m.p: 280 –81 °C; IR (KBr) cm^− 1^: 3100, 2924 (CH), 1718, (C = O), 1601 (C = N), 1545 (C = C); ^1^H NMR (DMSO-*d*_*6*_) δ 7.30- 8.00 (m, 15 H, Ar-H (, 8.40 (s, 1H, CH = N), 8.91 (s, 1H, CH-pyrazole), 9.19 (s,1H, CH-coumarin), 11.02 (s, 1H, NH); ^13^C NMR (DMSO-*d*_*6*_) δ 116.66- 158.56 (Ar-C), 164.52, 166.34 (2 C = O); MS *m/z* (%): 557 (M^+^, 3.47), 77 (100%). Anal. calcd. for C_31_H_19_N_5_O_4_S)557.58(: C, 66.78; H, 3.43; N, 12.56; S, 5.75%. Found: C, 66.69; H, 3.33; N, 12.64; S, 5.85%.

### 2-{N’-[3-(2-Oxo-2 H-chromen-3-yl)-1-phenyl-1 H-pyrazol-4-ylmethylene]-hydrazino}-5-(phenyl hydrazono)-thiazol-4-one (9)

Yellow powder; Yield: 70%; m.p: 270 –72 °C; MS *m/z* (%): 533 (M^+^, 2.70), 77 (100%). Anal. calcd. For C_28_H_19_N_7_O_3_S_2_)533.56): C, 63.03; H, 3.59; N, 18.38; S, 6.01%. Found: C, 63.09; H, 3.50; N, 18.31; S, 6.09%.

### 3-{4-[(4-Methyl-5-phenylazo-thiazol-2-yl)-hydrazonomethyl]-1-phenyl-1 H-pyrazol-3-yl}-chromen-2-one (10a)

Orange powder; Yield: 84%; m.p: 260 –61 °C; IR (KBr) cm^− 1^: 3427 (NH), 3052 (CH), 1721 (C = O), 1622 (C = N), 1596 (C = C); ^1^H NMR (DMSO-*d*_*6*_) δ 2.45 (s, 3 H, CH_3_), 7.38–7.65 (m, 12 H, Ar-H), 7.85 (t, 1H, *J* = 7.7 Hz, Ar-H), 7.95 (d, 1H, *J* = 7.6 Hz, Ar-H), 8.39 (s, 1H, CH = N), 8.93 (s, 1H, CH-pyrazole), 9.15 (s, 1H, CH-coumarin), 11.33 (s, 1H, NH). MS *m/z* (%): 533 (M^+^ + 2, 5.47), 532 (M^+^ + 1, 17.97), 531 (M^+^, 49.33), 77 (100). Anal. calcd. for C_29_H_21_N_7_O_2_S (531.59):C, 65.52; H, 3.98; N, 18.44; S, 6.03%. Found: C, 65.59; H, 3.92; N, 18.49; S, 6.09%.

### 3-{4-[(4-Phenyl-5-phenylazo-thiazol-2-yl)-hydrazonomethyl]-1-phenyl-1 H-pyrazol-3-yl}-chromen-2-one (10b)

Red powder; Yield: 80%; m.p: 160 –61 °C; IR (KBr) cm^− 1^: 3427 (NH), 3070, 2921 (CH), 1718 (C = O), 1602 (C = N), 1543 (C = C); ^1^H NMR (CDCl_3_) δ 7.29–7.80 (m, 19 H, Ar-H), 8.13 (s, 1H, CH = N), 8.55 (s, 1H, CH-pyrazole), 8.68 (s, 1H, CH-coumarin), 10.15 (s, 1H, NH); MS *m/z* (%): 593 (M^+^, 3.02), 77 (100%). Anal. calcd. for C_34_H_23_N_7_O_2_S (593.66): C, 68.79; H, 3.91; N, 16.52; S, 5.40%. Found: C, 68.72; H, 3.98; N, 16.60; S, 5.48%.

####  Cytotoxic assay

The experimental method used in anticancer screening has been adopted by the U.S. NCI according to reported standard procedure^55–57^.

#### NCI-60 screening methodology

The anticancer activity of the selected compounds was performed against different cancer cell lines at USA NCI according to the standard procedure. 23–25 Cell suspensions were diluted according to each cell type and the expected target cell density was added into microtiter plates Inoculates were allowed a pre-incubation period of 24 h at 37 °C for stabilization. Selected compounds were added at a concentration of (10 lmol) and cultures were incubated for 48 h at 37 °C in 5% CO_2_ atmosphere and 100% relative humidity. Estimation of cell viability or growth was done by Sulforhodamine B (SRB) protein assay. The results for one-dose data of each compound were reported as a mean graph which shows the percent growth of treated cells relative to untreated control cell and relative to the time zero number of cells. This allows the detection of both growth inhibition (values between 0 and 100) and lethality (values less than 0). This is the same as for the 5-dose assay. For example, a value of 100 means no growth inhibition; the value 45 would mean 55% growth inhibition. The zero value means no net growth over the course of the experiment. A value of − 40 would mean 40% lethality. A value of -100 means all cells are dead.

## Conclusion

A new series of coumarin derivatives were synthesized and investigated for their cytotoxic activity against 60 human cancer cell lines. The results of screening indicated that compounds **3a**, **3b** and **6** were the most active against almost cancer cell lines with IGP up to 96%. Moreover, compounds **6** and **10b** recorded lethal effect toward melanoma MDA-MB-435 with growth percent values − 47.47 and − 27.79%, respectively, also compound **6** had lethality effect against renal cancer A498 with growth percent values 6.20. Compound **10a** showed strong activity against melanoma MDA-MB-435 where the IGP values was 96.03%. .

## Data Availability

All data generated or analyzed are included in this published article and its supplementary information file.
